# Can we recruit to expertise trials? Experience from the total or partial knee arthroplasty trial (TOPKAT)

**DOI:** 10.1186/1745-6215-14-S1-O114

**Published:** 2013-11-29

**Authors:** Loretta Davies, Cushla Cooper, David Beard

**Affiliations:** 1 University of Oxford, Oxford, UK

## 

The difficulties of conducting a randomised controlled trial (RCT) of a surgical intervention have long been recognised. Individual surgeon skill and experience introduce an additional challenge to those of conventional RCTs and as a consequence, surgeons may be reluctant to participate in orthopaedic surgery trials.

The use of expertise based randomised trials has been proposed as an alternative design, where participants are randomised to surgeons with an expertise in the allocated intervention.

TOPKAT, a randomised controlled trial examining the clinical and cost effectiveness of total or partial knee replacements for medial compartment osteoarthritis utilised a combined expertise/equipoise based design in order to maximise surgeon participation and recruitment to the study. The trial is on-going and has currently enrolled 460/500 (92%) participants with recruitment due to end in September, 2013. Overall, there are twenty eight participating sites, twenty one of which are equipoise and remaining seven expertise sites.

Large variability in recruitment rates are seen across all sites with those participating as expertise sites demonstrating lower average monthly recruitment rates. Factors identified as additional challenges impacting recruitment rates and trial conduct to those of the equipoise sites include delays in establishing local expertise teams, patient preference for the recruiting surgeon, difficulties with patient logistics and timing of randomisation.

The TOPKAT trial demonstrates that recruitment of Orthopaedic surgeons to participate in and recruit to an expertise based design is both achievable and feasible. We highlight the potential challenges and possible considerations that may facilitate the conduct of expertise based trials.

